# Unleashing the power of DNA-encoded libraries for challenging targets in drug discovery

**DOI:** 10.1016/j.pscia.2026.100115

**Published:** 2026-03-10

**Authors:** Nana Du, Yijun Li, Qing Lou, Gong Zhang, Yangfeng Li, Yizhou Li

**Affiliations:** Chongqing Key Laboratory of Natural Product Synthesis and Drug Research, Innovative Drug Research Center, School of Pharmaceutical Sciences, Chongqing University, Chongqing, 401331, China

**Keywords:** DNA-Encoded library, Challenging targets, Drug discovery, Chemical space

## Abstract

DNA-encoded library (DEL) technology has emerged as a transformative platform in early-stage drug discovery, enabling the rapid and cost-effective exploration of ultra-large chemical spaces. However, identifying ligands for challenging targets characterized by featureless surfaces, high conformational plasticity, or shallow binding sites remains a formidable challenge. While the potential of DEL is widely recognized, a systematic evaluation of its strategic evolution against these intractable targets over the past fifteen years is timely. This review surveys the progress of DEL technology in tackling such targets, organized by GTPases, epigenetic regulators, phosphatases, protein–protein interaction (PPI), membrane proteins, and RNA. We highlight pivotal case studies and methodological breakthroughs while critically examining aspects of driving force in DEL such as DNA-compatible chemistry, diversified library design, advanced selection strategies, and artificial intelligence (AI) integration. Finally, we illustrate how DEL evolves from a conventional screening tool into a multifaceted discovery engine. By identifying future directions that include expanding three-dimensional chemical space, enhancing library fidelity, and deepening integration with functional biology and AI, this review provides a strategic roadmap to inspire and guide future DEL campaigns against those challenging targets.

## Highlights

• Challenging targets are biological targets that are difficult to modulate using conventional drug discovery approaches.
• This review presents case studies demonstrating DELs’ successful identification of ligands against challenging targets.
• Advances in chemical space, library design, selection methods, and AI will empower DEL to target challenging targets.

## Graphical abstract

**Figure:**
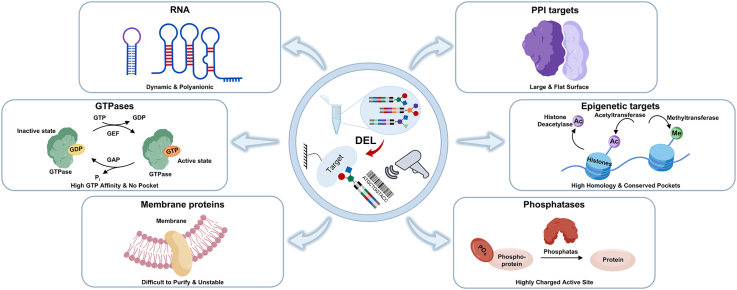

## 1 Introduction

With the rise of genomics and proteomics, an increasing number of disease-related targets have been identified [[1], [2], [3]]. However, only a small portion of these are considered “druggable” targets. A distinct class of biological macromolecules, characterized by large, complex, or functionally unique structures, remains difficult to modulate with traditional drug discovery methods. These are often called “undruggable” or “difficult-to-drug” targets. Research aiming at these challenging targets is now viewed as a major opportunity for therapeutic innovation, drawing significant attention and resources across the biomedical field. Typically, challenging targets share several features: (1) they possess extended and flat functional interfaces that lack deep and defined ligand-binding pockets; (2) they often have no known specific ligands capable of producing functional effects; (3) some are inhibitory or loss-of-function disease-related proteins, for which no ligands currently exist to restore or activate their function; and (4) their inherent chemical properties and conformational flexibility make designing small-molecule modulators particularly challenging. Traditional drug targets, such as kinases and ion channels, are not regarded as challenging targets in this review due to the relative ease of ligand discovery and the well-established methods. From the current perspective, challenging targets are generally classified into the following categories [3,4]: (1) GTPases, like the RAS family protein KRAS; (2) epigenetic targets; (3) phosphatases, such as ACP3; (4) PPI targets, such as anti-apoptotic Bcl-2 family members; (5) membrane proteins, and (6) RNA.

The development of drugs against these challenging targets has been hindered by the complexity of the targets and the limitations of conventional discovery methods. Although high-throughput screening (HTS) has been a key tool in drug discovery for many years, it has several drawbacks. These include the lack of target-focused compound libraries, the long timescales of screening, and the high costs associated with library construction, which make it difficult to explore the vast chemical space or effectively target complex biological entities. Advances in fragment-based drug discovery (FBDD), computer-aided drug design (CADD), virtual screening (VS), and DEL technology have provided new strategies and tools to tackle these challenges [5]. Among these, DEL has emerged as a powerful and low-cost approach for hit identification.

**Fig. 1:**
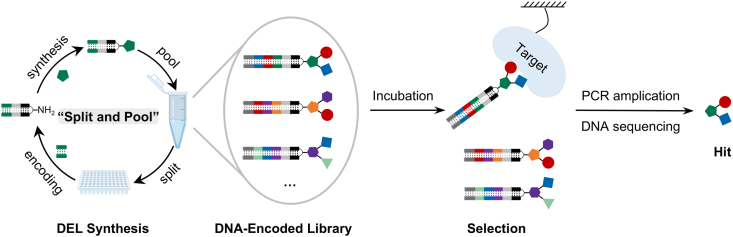
The classical workflow of DEL.

The concept of DEL was first proposed by Brenner and Lerner in 1992 [6] and subsequently realized experimentally by Nielsen et al., in 1993 [7]. In 2004, four pioneering solution-phase DEL formats were independently reported by Neri [8], Liu [9], Harbury [10], Winssinger [11], and their respective colleagues: namely, the dual-pharmacophore encoded self-assembling chemical library, the DNA-templated synthesis library, the DNA-routing library, and the peptide nucleic acid (PNA)-encoded library. A major milestone came in 2009, when GlaxoSmithKline (GSK) developed the first large-scale industrial application of DEL [12]. These foundational advances ushered in a period of rapid development for the technology. Specifically, DELs employ a “split-and-pool” synthesis strategy to covalently link diverse small molecules with unique DNA barcodes, thereby constructing ultra-large libraries encompassing billions of compounds. During the selection process, the DEL is incubated with an immobilized target. The enriched DNA barcodes are then PCR-amplified and sequenced to identify ligands with target-binding capabilities (Fig. 1). In contrast to conventional drug discovery methods, each library member in DELs is uniquely paired with a DNA barcode, which allows the entire compound library to be pooled together in a single test tube for simultaneous target-binding analysis. Furthermore, by leveraging PCR’s capacity to amplify DNA tags, the physical scale of selection is drastically reduced. At the same time, the availability of large-scale next-generation sequencing (NGS) enables DELs to achieve library sizes of up to billions of members. Consequently, DEL-based selection can typically be completed within days, requiring only tens of micrograms of a typical protein target and picomolar quantities of DEL samples. In summary, DEL uniquely combines massive experimental library scale, high-throughput validation, and direct access to high-quality, actionable chemical matter—making it a particularly powerful tool for challenging drug targets [13].

This review provides a systematic overview of the latest advances in applying DEL technology to challenging targets. We focus on strategic innovations and representative case studies in targeting PPIs, membrane proteins, RNA, and other non-classical binding targets. Furthermore, we discuss how key enabling technologies, including development of DNA-compatible chemistry, diversification of library design, advancement of selection strategies, and integration with AI, are driving DEL toward higher throughput and success rates. Finally, we highlight the transformative potential of DEL in future drug discovery and outline perspectives for its continued development.

## 2 Recent advances and applications for DEL in addressing challenging targets

### 2.1 GTPases

GTPases constitute a large family of enzymes with relatively low molecular weights that are capable of hydrolyzing GTP. This family encompasses over 150 distinct members, which can be categorized into five subfamilies based on sequence homology and physiological functions: the Rat Sarcoma (Ras) family, the Rhodopsin (Rho) family, the Ras-related in brain (Rab) family, the ADP-ribosylation factor (Arf) family, and the Ras-related nuclear protein (Ran) family. GTPases mediate cellular processes through recycling between the GTP-bound state “active” and the GDP-bound state “inactive” [14]. They play an essential role in regulating a diverse range of cellular activities [15,16], including cell survival, cell cycle progression, proliferation, apoptosis, differentiation, adhesion, and migration [14,17], as well as subcellular events such as the regulation of the actin cytoskeleton and vesicle trafficking [18,19]. However, the GTPase family has long been considered a challenging therapeutic target due to its picomolar affinity for GTP and the absence of well-defined binding pockets on its protein surface. Recently, significant progress has been made in the pharmacological targeting of one of the more frequent activating KRAS mutations, KRAS^G12C^, in which glycine at codon 12 is substituted by cysteine (G12C). This cysteine substitution renders KRAS^G12C^ susceptible to covalent inhibition by small-molecule inhibitors equipped with cysteine-reactive electrophilic warheads. The formation of a covalent bond between the inhibitor and Cys12 locks KRAS^G12C^ in the GDP-bound, inactive state. The success of this targeted covalent inhibition strategy is evidenced by the FDA-approved KRAS^G12C^ inhibitors, sotorasib and adagrasib [20].

**Fig. 2:**
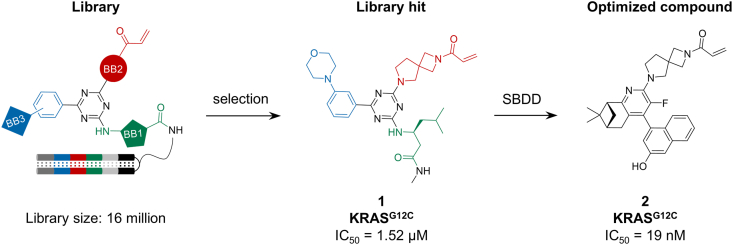
Ligands targeting the GTPase KRAS^G12C^.

In February 2025, Amgen reported a KRAS^G12C^ covalent inhibitor. Researchers leveraged recently reported information on covalent inhibitors targeting KRAS^G12C^ to construct a focused covalent DEL tailored for KRAS^G12C^. This library, centered around a triazine core composed of three substituents, with one capped by a Cys-reactive acrylamide functional group, encompassed approximately 16 million members. Through two sets of counterscreening assays utilizing a KRAS^G12V^ counterscreen and a “beads-only” control, a series of potential hits were identified (such as compound **1**, IC_50_ = 1.52 μM, Fig. 2). Subsequently, by integrating structure-based drug design (SBDD) and structure-activity relationship (SAR) optimization, a novel and highly potent covalent ligand **2** targeting KRAS^G12C^ was discovered, exhibiting an IC_50_ of 19 nM (Fig. 2). This work demonstrates the application of covalent DEL selection in addressing challenging targets [21].

### 2.2 Epigenetic targets

“Epigenetics” refers to stable and heritable phenotypic changes that occur without alterations to the DNA sequence, resulting from chromosomal modifications [22]. It regulates chromatin state via five mechanisms: DNA, histone, RNA modification, and chromatin remodeling. Over decades, epigenetic mutations (abnormal DNA methylation, histone modifications, and altered chromatin structures) have played key roles in various diseases, especially tumor initiation [23], invasion, metastasis [24], immune evasion [25], and drug resistance [26]. Therefore, epigenetic regulation is an attractive drug-development strategy for diseases like cancer [27,28]. However, discovering potent and selective inhibitors for catalytic sites is challenging due to high sequence homology and conserved catalytic core pockets in the same epigenetic enzyme subfamily. Additionally, epigenetic modifications are carried out by multi-protein complexes, where the activation of catalytic subunits is strictly regulated, making ligand development challenging [29]. In this context, DEL, with its capacity to construct ultra-large yet focused chemical collections, emerges as a powerful platform to address these challenges, enabling the discovery of high-affinity and selective ligands against such elusive targets.

**Fig. 3:**
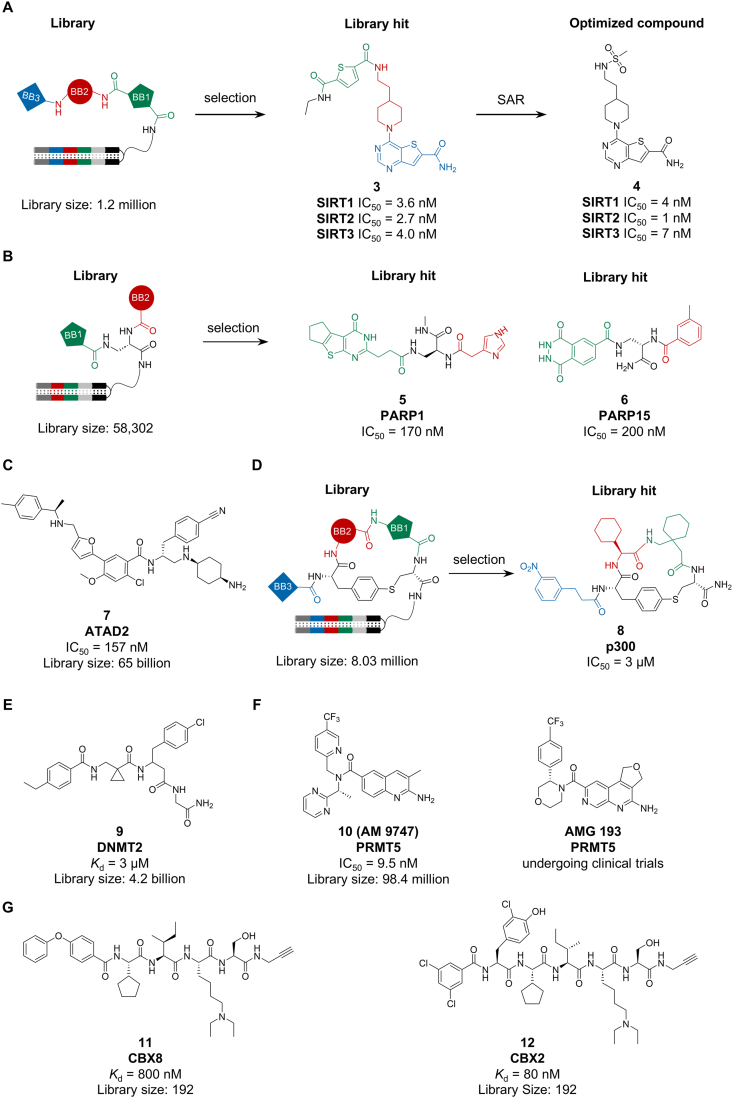
Ligands targeting the epigenetic targets (A) SIRT1, SIRT2 and SIRT3; (B) PARP1 and PARP15; (C) ATAD2; (D) p300; (E) DNMT2; (F) PRMT5; (G) CBX8 and CBX2.

The sirtuins SIRT1, SIRT2, and SIRT3 are NAD^+^ dependent deacetylases, recognized as potential therapeutic targets for metabolic [30], inflammatory, oncologic [31], and neurodegenerative disorders. In 2013, researchers from GSK and their collaborators used a heterocycle-enriched DEL to discover pan-inhibitors of SIRT1/2/3 with nanomolar potency. The initial hit **3** showed IC_50_ values of 3.6 nM (SIRT1), 2.7 nM (SIRT2) and 4.0 nM (SIRT3). SAR studies yielded drug-like analogue **4** with improved potency (IC_50_ = 4 nM, 1 nM and 7 nM, respectively) [32] (Fig. 3A). In 2019, Franzini et al. constructed a DEL designed for NAD^+^-binding pockets (NADEL), utilizing a 2,3-diaminopropanamide scaffold to assemble two fragments that mimic the overall shape of a cofactor. The selection information guided the synthesis of inhibitors for various enzymes, including PARP1 (compound **5**, IC_50_ = 170 nM), and PARP15 (compound **6**, IC_50_ = 200 nM) (Fig. 3B). Comparison with other libraries confirmed that target-complementary design enhanced selection efficiency [33].

ATPase family AAA-domain containing protein 2 (ATAD2), an epigenetic regulator and transcriptional co-factor, is overexpressed in the progression of various cancers [34]. In a 2017 study, ATAD2 was chosen as a target by researchers from X-Chem, who conducted a DEL selection encompassing 65 billion members (11 DELs). This effort led to the discovery of compound **7**, a potent and isoform-selective inhibitor (IC_50_ = 157 nM, Fig. 3C) that specifically induces ATAD2 bromodomain dimerization, disrupts its interactions with acetylated histones in vitro, and with chromatin in cells, thereby qualifying it as a valuable chemical probe for exploring ATAD2 biology [35].

E1A-binding protein p300 and its paralog CREB-binding protein (CBP) are key histone acetyltransferases (HATs) involved in transcriptional regulation. Aberrant p300 activity is implicated in cancers, making it a promising therapeutic target [36,37]. In 2021, Lu and Chen developed a mild, palladium-catalyzed intramolecular S-arylation method to construct cyclic peptides and prepared an 8-million-member cyclic peptide DEL. Screening this library against p300 identified compound **8** (IC_50_ = 3 μM, Fig. 3D) [38]. Subsequently, Lu et al. built a DEL based on an indazolone core via a DNA-compatible light-promoted reaction and discovered additional p300-targeting ligands [39].

Targeting the RNA methyltransferase DNMT2 is challenging due to poor selectivity of known SAH-derived ligands [40]. Barthels et al. screened a DEL and identified five non-SAH chemotypes with unprecedented selectivity. Optimization afforded **9** (*K*_d_ = 3 μM, Fig. 3E), providing both selective scaffolds for therapeutics and insights into DNMT2 allostery and structural plasticity [41].

Protein arginine methyltransferase 5 (PRMT5), through methylation [42], plays a pivotal role in regulating various cellular processes, including chromatin remodeling and gene expression [43,44]. Notably, elevated expression levels of PRMT5 have been observed in various cancers, and this high expression is correlated with poor prognosis and tumor metastasis. Inhibition of the methyltransferase enzyme PRMT5 by MTA accumulation is a vulnerability of MTAP-deleted cancers. Endogenous PRMT5 forms a heterooctameric complex with methylosome protein 50 (MEP50). In 2025, Amgen selected a 98.4-million-member DEL and identified **AM 9747** (compound **10**, IC_50_ = 9.5 nM, Fig. 3F) that could cooperatively bind PRMT5: MEP50 and MTA. This compound selectively inhibits PRMT5-mediated symmetric dimethylation and significantly reduces the viability of MTAP-deleted cells [45]. This work established the foundation for discovering **AMG 193** (Fig. 3F) [46], the first MTA-cooperative PRMT5 inhibitor undergoing Phase I/II clinical trials (NCT05094336, NCT05975073) for the treatment of MTAP-deleted cancers.

Polycomb group (PcG) proteins, such as the CBX family (CBX2/4/6/7/8), regulate gene expression via conserved N-terminal chromodomains (ChDs) that recognize H3K27me3 [[47], [48], [49], [50], [51]]. The high structural similarity among CBX ChDs complicates the development of selective inhibitors. In 2020, Dykhuizen et al. constructed a focused DEL based on a known CBX8 ChD inhibitor. Through iterative selection that involved first assessing selectivity across CBX paralogs and then quantifying affinity for CBX8, they identified a cell-permeable peptidomimetic ligand for CBX8 ChD (compound **11**, *K*_d_ = 800 nM, Fig. 3G). This work exemplified the use of DEL for simultaneous optimization of potency and selectivity [52]. Building on this approach, further optimization in 2021 yielded a potent and selective CBX2 ChD inhibitor (compound **12**, *K*_d_ = 80 nM, Fig. 3G), demonstrating that DELs can overcome high homology to generate paralog-selective chemical probes [53].

### 2.3 Phosphatases

Phosphatases serve as key regulators in cellular dynamics, exerting their function by catalyzing the dephosphorylation of proteins at specific residues, namely serine, threonine, and tyrosine [54]. According to their structural characteristics, phosphatases can be classified into two categories: protein tyrosine phosphatases (PTPs) and protein serine/threonine phosphatases (PSTPs) [3]. Phosphatases hold significant importance in physiological regulation, as well as in severe pathological conditions, including malignant tumors, diabetes, asthma, cystic fibrosis, immunosuppression, and cardiovascular diseases [55]. Unfortunately, the high structural similarity within each category of phosphatases leads to low selectivity, inevitable off-target effects, and the preference of their active sites for negatively charged compounds [56,57], which poses challenges in terms of bioavailability. These factors have substantially hindered progress in drug discovery. To address the aforementioned challenges, a strategy of targeting allosteric sites can be employed to discover highly selective hit compounds, followed by rational structural optimization to enhance membrane permeability and drug-like properties.

**Fig. 4:**
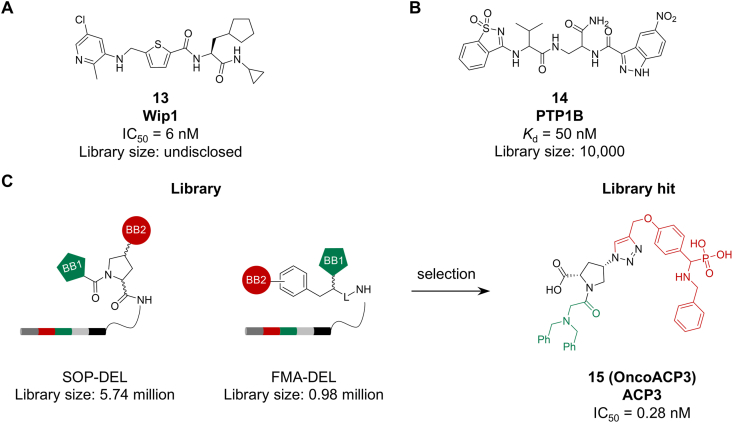
Ligands targeting phosphatases (A) Wip1; (B) PTP1B; (C) ACP3.

Wild-type p53-induced phosphatase (Wip1) is an oncogenic serine/threonine phosphatase whose highly conserved catalytic domain hinders the development of selective inhibitors [58]. To address this challenge, GSK combined a DEL selection against full-length Wip1 with a biochemical HTS assay using a truncated Wip1. The DEL selection yielded a potent inhibitor, while HTS identified hits with overlapping structural features. Through rational optimization of cell permeability and pharmacokinetics, compound **13** was developed as the first orally active, allosteric Wip1 inhibitor with an IC_50_ of 6 nM (Fig. 4A). This work demonstrates how affinity-based selection can identify allosteric binders, offering a strategy for targeting this challenging enzyme class [59].

Protein tyrosine phosphatase 1B (PTP1B) is a key therapeutic target, implicated in insulin sensitization and breast cancer [60]. In 2016, Winssinger et al. utilized DNA display of PNA-encoded libraries to screen 62,500 combinatorial fragment pairs against PTP1B. Selected fragments were then linked with diverse spacers to construct a focused DEL. Screening against both PTP1B and the closely related TCPTP yielded orthogonal inhibitors, with compound **14** (*K*_d_ = 50 nM, EC_50_ = 250 nM, Fig. 4B) showing potent and selective inhibition of PTP1B [61].

While radioligand therapy (RLT) targeting prostate-specific membrane antigen (PSMA, e.g., Pluvicto) is clinically approved, its off-target uptake in healthy tissues remains a concern [62]. Prostatic acid phosphatase (ACP3) has emerged as a promising alternative target, showing high expression in prostate cancer with minimal presence in healthy organs [63]. In 2025, Philochem reported the discovery of ACP3 ligands using DEL technology. Two DELs, SOP-DEL (proline-based) and FMA-DEL (phenylalanine-based), were screened against purified human ACP3. This effort identified compound **15** (**OncoACP3**) as a high-affinity ligand with an IC_50_ of 0.28 nM (Fig. 4C). The ligand was further engineered into RLT agents and small molecule-drug conjugates (SMDCs) for prostate cancer therapy. Several **OncoACP3**-derived radioligands are now in preclinical development, with **^68^Ga-OncoACP3** advancing to Phase I trials. This study underscores the capability of DEL to deliver ultra-high-affinity (pM-level) tumor-targeting ligands, establishing a blueprint for ligand discovery against other cancer targets [64].

### 2.4 PPI targets

PPIs are fundamental to nearly all biological processes and serve as core mechanisms for cellular signal transduction, metabolism, and gene regulation [65]. The human interactome encompasses an extensive network of PPIs, estimated to range from 130,000 to 650,000 distinct interactions [66]. Dysregulation of PPIs contributes to diseases such as cancer, neurodegeneration, and infectious diseases, establishing them as promising therapeutic targets [[67], [68], [69], [70]]. Despite their therapeutic appeal, targeting PPIs remains challenging due to the unique physicochemical properties of their interaction interfaces. These interfaces are typically large (1500–3000 Å), hydrophobic, and flat, lacking deep binding pockets required for conventional small-molecule binding [71]. Moreover, the high-affinity residue networks at these interfaces are difficult to disrupt with low-molecular-weight compounds [72]. To address these obstacles, diverse modulation strategies have emerged. Traditional approaches include small molecules, macrocycles, and peptides that directly inhibit interaction interfaces. Recently, targeted protein degradation (TPD) technologies such as PROTACs and molecular glues have provided a catalytic, event-driven alternative for challenging PPIs [68,73]. The following cases highlight key advances across these strategic categories.

**Fig. 5:**
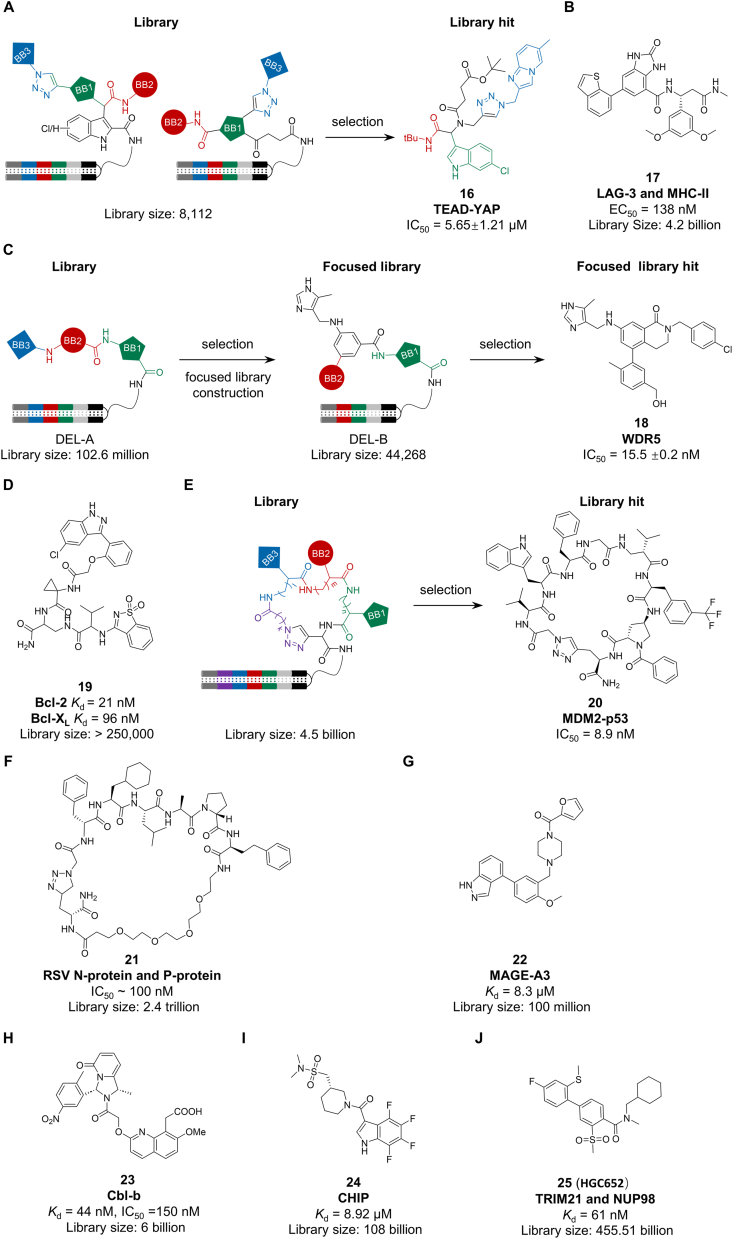
Ligands targeting PPI targets (A) TEAD-YAP; (B) LAG-3 and MHC-II; (C) WDR5; (D) BCL-2 and BCL-X_L_; (E) MDM2-p53; (F) RSV N-protein and P-protein; (G) MAGE-A3; (H) CBL-B; (I) CHIP; (J) TRIM21 and NUP98.

Small molecules are well-suited for targeting PPI interfaces due to their compact size and favorable physicochemical properties [74]. DEL technology has proven to be a powerful platform for discovering such PPI modulators. For example, a focused DEL of 8,112 compounds designed around the Ugi four-component reaction yielded a TEAD–YAP inhibitor (compound **16**, IC_50_ = 5.65 μM, Fig. 5A) [[75], [76], [77]]. Notably, TEAD is not only a key component in the PPI system but also serves as an epigenetic target, highlighting the multifaceted significance of the discovered inhibitor in both PPI regulation and epigenetic research. Screening of a 4.2-billion-member DEL identified a sub-micromolar ligand (compound **17**, Fig. 5B) that disrupts the LAG-3/MHC-II immune checkpoint interaction [78]. DEL also supports advanced optimization strategies: a cascade selection approach against WDR5–MLL1 generated a potent inhibitor (compound **18**, IC_50_ = 15.5 nM, Fig. 5C) [[79], [80], [81]], while a fragment-based DEL strategy enabled the discovery of a dual BCL-X_L_/BCL-2 inhibitor (compound **19**, Fig. 5D) with cytotoxicity comparable to venetoclax [82]. Together, these examples illustrate the versatility of DEL in PPI drug discovery, spanning from de novo hit identification to lead optimization via iterative and fragment-based approaches.

Macrocyclic compounds exhibit enhanced stability, cell permeability, binding affinity, and oral bioavailability, making them ideal for targeting PPIs [65,83]. This approach has been successfully implemented using DEL technology. For instance, to inhibit the oncogenic MDM2–p53 interaction [84], Silvestri et al. constructed a cyclic peptide DEL of 4.5 billion members via CuAAC-mediated macrocyclization and identified a potent inhibitor (compound **20**, IC_50_ = 8.9 nM, Fig. 5E) with favorable cell-permeability profiles [85]. Similarly, a trillion-member macrocyclic peptide DEL targeting the respiratory syncytial virus N–P protein interaction yielded a potent disruptor (compound **21**, IC_50_ ∼ 100 nM, Fig. 5F) [86]. Structural studies confirmed that macrocyclization improved binding affinity by 3–8 fold over linear analogs, highlighting the thermodynamic benefit of conformational constraint in PPI inhibition [87]. These cases illustrate that screening rationally designed macrocyclic DEL can efficiently deliver PPI inhibitors with high potency, desirable drug-like properties, and clear mechanistic insights.

Furthermore, DEL is proving invaluable for discovering ligands that enable more complex PPI-modulating modalities, such as PROTACs and molecular glues, which often require rare E3 ligase binders. For example, Crews et al. used DEL to identify a MAGE-A3 ligand (compound **22**, Fig. 5G) that was subsequently developed into a tumor-selective PROTAC, demonstrating a full workflow from ligand discovery to functional degrader [88]. Similarly, screening of an approximately 6-billion-compound DEL yielded a potent SH2-domain ligand for Cbl-b (compound **23**, *K*_d_ = 44 nM, IC_50_ = 150 nM, Fig. 5H) that disrupts its target PPI [89]. To address the historic lack of drug-like ligands for CHIP, a DEL of 108 billion compounds was screened, delivering the most potent small-molecule CHIP binder to date (compound **24**, *K*_d_ = 8.92 μM, Fig. 5I) as a starting point for PROTAC development [90,91]. Most notably, DEL enabled the discovery of a TRIM21 ligand that was advanced into a molecular glue degrader (compound **25**, **HGC652**, Fig. 5J), which redirects TRIM21 to degrade nuclear pore complex proteins [92]. These cases underscore DEL’s high throughput and success rate in accelerating the development of next-generation PPI modulators, including PROTACs and molecular glues.

### 2.5 Membrane proteins

Membrane proteins, which perform a myriad of biological functions, are critically important in human health and disease, constituting over 60% of the targets for approved small-molecule drugs [93]. Notable examples include G protein-coupled receptors (GPCRs) and ion channels, which alone account for approximately 34% and 18% of all clinical drugs [94,95]. However, ligand discovery for membrane proteins remains exceptionally challenging. Residing within the hydrophobic lipid bilayer of the cell membrane, these proteins pose significant challenges for in vitro biochemical techniques, including expression, solubilization, purification, and crystallization. Moreover, once purified, membrane proteins may lose vital biological features, including post-translational modifications, cofactor binding, and native complex formation [93,96]. Consequently, there is a compelling need to develop ligand discovery methods that are compatible with intact living cells, enabling the study of these targets within their native physiological context.

**Fig. 6:**
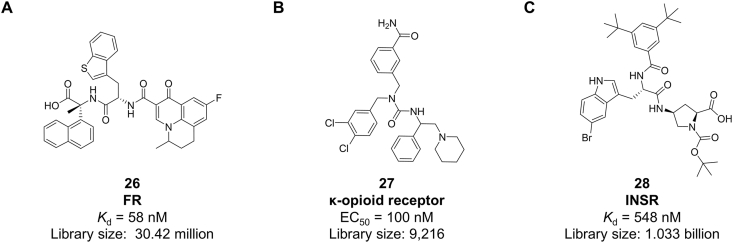
Ligands targeting Membrane proteins (A) FR; (B) κ-opioid receptors; (C) INSR.

DEL selection has been successfully extended to membrane proteins on live cells, overcoming challenges associated with protein purification and tag interference. Early examples include selections against the NK3 receptor and opioid receptors on live cells [97,98]. A significant advance was reported by Li et al., who developed a site-specific DNA labeling strategy that enables DEL selection of endogenous membrane proteins without overexpression or genetic modification. By tagging target proteins with DNA to facilitate localized DEL hybridization and enrichment, the method preserves native conformation and physiological context. Screening of a 30.42-million-compound DEL against three endogenous targets—folate receptor (FR), carbonic anhydrase 12 (CA12), and epidermal growth factor receptor (EGFR)—yielded novel ligands, including the FR binder with a *K*_d_ of 58 nM (compound **26**, Fig. 6A) [99]. This approach demonstrates the potential of DEL to directly probe endogenous membrane proteins in their native cellular environment, accelerating ligand discovery for this challenging target class.

In 2023, the Krusemark group employed a split protein complementation approach combined with DEL to conduct a selection on living cells. This method enabled the direct identification of DNA-linked molecules capable of inducing the dimerization of two proteins. Applied to δ-, μ-, and κ-opioid receptors, this approach led to the discovery of a selective, G-protein-biased agonist of the κ-opioid receptor (compound **27,** Fig. 6B), which exhibited an EC_50_ of 100 nM [100].

In 2024, Li reported a method for directly identifying small-molecule agonists of membrane proteins by screening DEL on live cells. This approach functionally couples the extracellular event of ligand binding to an intracellular biochemical cascade, thereby enabling a positive selection strategy for agonist discovery. Ultimately, an agonist (compound **28**, Fig. 6C) targeting the insulin receptor (INSR) was obtained, with a dissociation constant of 548 nM. This study developed a “functional” DEL selection strategy for cell-surface targets, paving the way for a broadly applicable approach to agonist discovery for membrane proteins [101].

### 2.6 RNA

The ENCODE project has revealed that a substantial portion of the human genome is transcribed into RNA, with only a limited proportion (approximately 1.5%) encoding proteins [102]. While the mainstream paradigm in drug discovery has traditionally focused on identifying and targeting disease-associated proteins, this approach is increasingly constrained by the limited number of protein targets and their druggability [103,104]. As RNA lies upstream of proteins and controls their translation, targeting RNA in drug research can, to some extent, address the challenge of “undruggable” proteins. Furthermore, some RNAs are directly implicated in diseases, such as long non-coding RNAs [105,106], microRNAs [107,108], and riboswitches [[109], [110], [111], [112]]. Currently, RNA targeting has emerged as a burgeoning direction in drug discovery. Drug development targeting RNA primarily follows several technological pathways: oligonucleotide therapies based on the principle of base-pair complementarity, such as antisense oligonucleotides (ASOs) [113] and small interfering RNAs (siRNAs) [114,115]; precise manipulation of RNA using gene editing systems [116,117] (e.g., CRISPR/Cas); the exploration of small-molecule drugs capable of specifically recognizing and binding to RNA structures [118]. Compared to the aforementioned macromolecular therapies, small-molecule drugs offer advantages such as superior cell membrane permeability, excellent oral bioavailability, extensive tissue distribution, stable physicochemical properties, and flexible chemical modification space [119]. This has made the development of small-molecule ligands targeting RNA a research hotspot. In August 2020, Risdiplam, jointly developed by Roche, PTC Therapeutics, and the SMA Foundation, was approved by the FDA for the treatment of spinal muscular atrophy (SMA) [120]. This marked the successful introduction of the first selective small-molecule drug targeting RNA, demonstrating the potential of RNA-targeted small molecules and serving as a pivotal milestone in the field’s development.

Despite the promising momentum in the development of small-molecule drugs targeting RNA, challenges persist: First, the highly negatively charged backbone of RNA restricts the number of compatible molecular structures. Second, compared to proteins composed of 20 amino acids, RNA has only four nucleobases, resulting in lower structural specificity and discernibility, which limits drug selectivity. Third, the flexibility of RNA structures makes it difficult for most RNAs to form stable conformations in vitro, and RNA molecules bind to numerous proteins, posing significant challenges for RNA structural elucidation and ligand discovery [121]. To date, affinity-screening-mass-spectrometry (AS-MS) [122,123] and FBDD [124] represent the most prevalent target-based screening approaches for purified RNA. However, the limited size of fragment libraries, coupled with the historical bias of HTS compound collections towards protein interactions rather than RNA interactions, has constrained the efficiency of discovering novel ligands targeting RNA. Fortunately, DEL, which boasts unique advantages in terms of vast chemical diversity, privileged scaffold libraries and multiple affinity selection capabilities, has achieved some notable successes in screening for RNA targets.

**Fig. 7:**
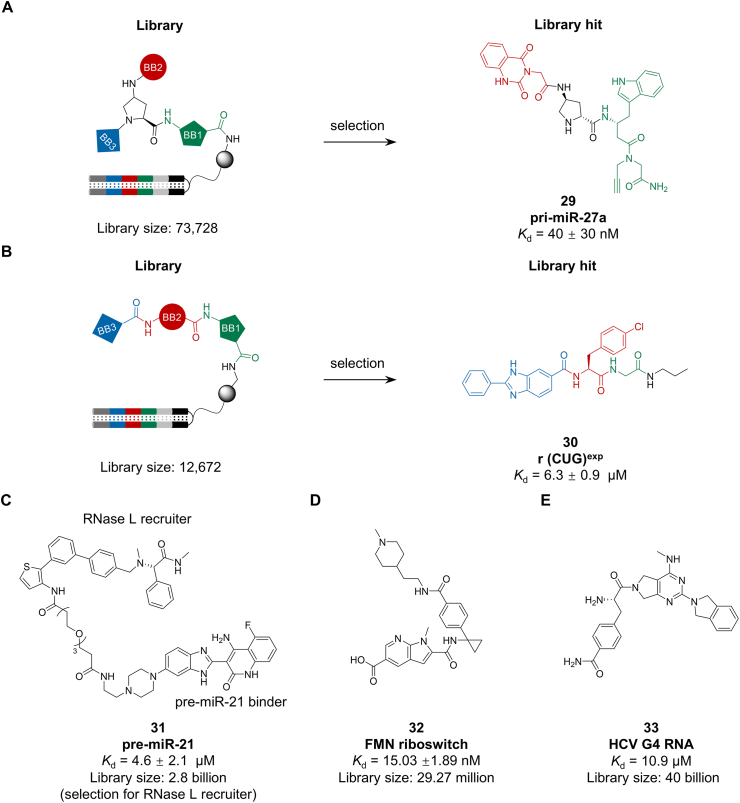
Ligands targeting RNA (A) pri-miR-27a; (B) r(CUG)^exp^; (C) RNase L; (D) FMN riboswitch; (E) HCV G4 RNA.

In 2022, the Disney group established an integrated screening platform combining two-dimensional combinatorial screening (2DCS) with solid-phase DEL technology to systematically profile RNA–small molecule interactions. A solid-phase DEL of 73,728 compounds, constructed from a trifunctional amino acid scaffold (Fmoc-Pro(N_3_)-OH), was screened against an RNA library of 4096 variants containing a conserved 3 × 3 nucleotide internal loop motif. Using fluorescence-activated cell sorting (FACS), approximately 300 million molecular interactions were evaluated, enabling the identification of several ligand–RNA pairs with structural specificity. Among these, compound **29** demonstrated nanomolar affinity (*K*_d_ = 40 ± 30 nM, Fig. 7A) for oncogenic pri-miR-27a, leading to inhibition of miRNA biogenesis and suppression of cell migration in triple-negative breast cancer models. This approach advances beyond conventional single-target screening by mapping interaction landscapes in a massively parallel manner, offering a generalizable strategy for targeting RNA structural motifs in drug discovery [125].

Expanded r(CUG) repeats in the 3’UTR of the DMPK mRNA form structured hairpins that sequester the splicing regulator MBNL1, driving pathogenesis in myotonic dystrophy type 1 (DM1) [126]. In 2022, Disney and colleagues employed a DEL strategy to target this RNA. They constructed a solid-phase DEL of 12,672 compounds using Fmoc-protected amino acids and nitrogen-rich heterocycles. Screening via two-color flow cytometry identified uncharged, selective binders for r(CUG)^exp^. The lead molecule (compound **30**, Fig. 7B) not only bound the pathogenic RNA but also ameliorated DM1-associated translational defects. Further conversion of this binder into a targeted degrader enhanced its efficacy by selectively cleaving the toxic transcripts, improving cellular DM1 phenotypes [127]. This work exemplifies how DEL can deliver functional RNA-binding small molecules and illustrates the potential of coupling DEL-discovered binders with degradation modalities for therapeutic RNA targeting.

In the same year, Disney et al. adopted the DEL approach to identify a binder for the monomer RNase L, which induces RNase L dimerization and activates its ribonuclease activity. Using this compound as a starting point, the next-generation RiboTAC was designed (compound **31**, Fig. 7C). It targets the miR-21 precursor (pre-miR-21) and is capable of alleviating miR-21-associated cellular phenotypes in triple-negative breast cancer cells [128].

In 2022, HitGen reported the discovery of flavin mononucleotide (FMN) riboswitch ligands via DEL selection. Initial efforts against the HIV-1 TAR RNA highlighted a major challenge: high background from nonspecific DNA–RNA interactions. To address this, the team developed an optimized selection strategy combining RNA patches and competitive elution to suppress off-target binding, supplemented by computational k-mer and motif analysis to distinguish true hits. Applying this approach to a 46.3-billion-member “super-DEL” validated DEL’s feasibility for structured RNA targets. Subsequent screening of a focused sub-library of 29.27 million compounds yielded two lead molecules with nanomolar affinity (*K*_d_ = 12–15 nM) and potent inhibitory activity, outperforming reference inhibitors by 2–3 fold in biochemical assays (e.g., compound **32**, Fig. 7D). This work not only expands the utility of DEL to RNA targets but also establishes a robust selection and analysis framework for mining specific ligands within ultra-large chemical spaces [129].

The G-quatroplex (G4) of the Hepatitis C Virus (HCV), located in the 5’ or 3’ UTRs of its mRNA, is a short yet structured viral RNA composed of stacked planar G-quadruplexes, representing a potential drug target. In 2025, the researchers from WuXi AppTec disclosed a small molecule that binds to this RNA target. Initially, a fully double-stranded “DEL Zipper” library targeting THF-riboswitch RNA was constructed innovatively, along with an RNA-specific motif warning workflow. Subsequently, they employed the “DEL Zipper” library, which contains 157 DEL libraries with more than 40 billion diversity molecules, to screen for HCV G4 RNA. The hit compounds were identified through this selection and underwent off-DNA validation. Notably, the ligand **33** exhibited a *K*_d_ of 10.9 μM against the target wild-type G4-RNA (Fig. 7E). Overall, this work demonstrates that the “DEL Zipper” based selection methodology can be effectively applied to structured RNA targets [130].

## 3 Innovative development of DEL technology to target unexplored challenging targets

### 3.1 Developing DNA-compatible chemistry

DNA-compatible chemistry serves as the key enabler for DEL technology, directly governing the diversity of the chemical space, the drug-likeness of library members, and the feasibility of sophisticated library designs. This foundational role necessitates that all on-DNA syntheses proceed under mild, dilute aqueous conditions, achieving high conversion and broad substrate compatibility while avoiding factors detrimental to DNA integrity, such as high temperatures and strong oxidants. Without DNA-compatible chemistry, the realization and expansion of DEL technology would be unattainable. The initial development of DELs was constrained by a limited DNA-compatible chemistry toolkit, primarily reliant on amidation and click chemistry, which restricted structural diversity. Continuous methodological innovations, however, have dramatically expanded this toolbox, enabling the construction of libraries featuring structurally complex and drug-like molecules [131].

On the one hand, the development of methodologies such as heterocycle formation, macrocyclization, and functional group transformations (FGTs) has significantly expanded the accessible chemical space. For example, heterocyclic scaffolds serve as privileged frameworks for constructing drug-like libraries, owing to their broad bioactivity, structural diversity, and strong binding affinity toward target proteins. This includes scaffolds such as benzimidazole [132], dihydroquinazolinone [133], dihydrothiazole [134], isoindole [135,136], isoindolin-1-imine [135], indazolone [135], and thiopyrrole [137]. Macrocyclization enhances binding affinity, particularly for challenging targets like PPI interfaces, by imposing rigid conformational constraints. Cyclization strategies include direct cyclization [38,[138], [139], [140]] and the grafting of the aforementioned privileged scaffolds. Furthermore, FGTs [[141], [142], [143], [144], [145], [146], [147], [148]] convert inert functional groups into reactive handles, enabling site-specific diversification and the one-step construction of drug-like privileged structures, thereby optimizing binding affinity and pharmacokinetics. DNA-compatible chemistry continues to hold significant potential for further development. For instance, to target biomolecules such as RNA, the introduction of specialized chemical motifs that include semi-fused ring systems and positively charged functional groups is essential to enhance the interaction capabilities of library members with biological macromolecules. Looking ahead, the integration of enzymatic catalysis with DEL technology holds promise for broadening the range of accessible reaction types and structural diversity [149], enabling precise modifications that are challenging to achieve through conventional organic synthesis methods. New types of DNA-compatible click reactions continue to emerge. For example, the Se-N exchange (SeNEx) click chemistry developed by Hou and Xu et al. enables efficient C–Se bond formation between benzoselenazolones and terminal alkynes under DNA-compatible conditions, offering a novel tool for the discovery of selenium-containing drugs [150]. The development of numerous other types of DNA-compatible chemical reactions is progressively enhancing the diversity of chemical space [[151], [152], [153], [154]].

On the other hand, chirality and sp^3^-hybridized carbons represent indispensable stereochemical elements in drug development. Studies indicate that over the past decade, approximately 60% of FDA-approved drugs have been chiral, with this proportion continuing to rise steadily [155]. The high proportion of approved chiral drugs underscores the critical importance of stereochemical control, while three-dimensional structures rich in sp^3^-carbons significantly enhance drug-like properties, target-binding specificity, and metabolic stability [156]. To effectively access and explore this privileged chemical space within DEL, there is an urgent need to develop DNA-compatible synthetic methodologies capable of efficiently introducing chiral centers and sp^3^-hybridized carbons, such as asymmetric catalysis and enzymatic catalysis, thereby addressing the demands of modern drug discovery targeting complex biological targets. In summary, the development of DNA-compatible chemistry is the core driver behind the transition of DEL technology from concept to practical application. The future development of DNA-compatible chemistry holds significant potential, with its core focus shifting from merely expanding reaction types toward the precise modulation of molecular properties and functional integration.

### 3.2 Diverse DEL design

Recent advancements in DEL design have transformed the technology from a broad, empirical screening tool into a precision-driven platform, enhancing ligand discovery efficiency [157]. Innovations such as fragment-based DEL [[158], [159], [160], [161], [162], [163]] enable systematic exploration of chemical space with minimalist building blocks, allowing for the efficient discovery of diverse and structurally novel ligands. For instance, by employing DNA-templated PNA libraries and dynamic libraries of dual-display formats, the Winssinger group discovered novel ligands targeting carbonic anhydrases, phosphatase PTP1B [61], and Bcl-X_L_/Bcl-2 [82]. Covalent DELs [11,[164], [165], [166]], which incorporate covalent warheads to facilitate irreversible binding to specific residues, enhance the efficiency of discovering potent inhibitors by capturing targets with high affinity. As a case in point, researchers from Amgen developed a focused covalent DEL and discovered a KRAS^G12C^ inhibitor from it [21]. Additionally, focused DEL [33,81] tailored to protein families or RNA reduces off-target interactions and increases hit quality [167]. This strategy has proven effective across diverse target classes, yielding r(CUG)^exp^ ligands from an RNA-focused DEL [127], p300 inhibitors from an indazolone-focused DEL [39], and TEAD-YAP/MDM2 inhibitors from indole-focused Ugi peptidomimetic libraries [77]. Together, these strategies will significantly enhance DEL’s effectiveness, boosting the likelihood of identifying high-affinity, biologically relevant ligands and accelerating drug discovery. Building upon the success of fragment-based, covalent, and focused DEL, future efforts will increasingly focus on constructing specialized libraries following similar design principles. This expansion is expected to yield a growing number of drug candidates with superior potency and selectivity against challenging therapeutic targets.

### 3.3 Advancing selection strategies

In recent years, significant progress has been made in DEL selection strategies, with technical advancements primarily reflected in two dimensions: the diversification of screening targets and the increased complexity of screening environments.

In terms of target scope, DEL technology has successfully expanded beyond traditional purified proteins to more challenging targets, such as nucleic acids [125,127,129,130]. For example, researchers from HitGen conducted a DEL selection against the FMN riboswitch. By implementing optimized strategies, including RNA patches and competitive elution, they ultimately identified lead compounds with nanomolar affinity and potent inhibitory activity from a super-DEL containing 46.3 billion molecules [129]. In another example, researchers at WuXi AppTec innovatively constructed a double-stranded “DEL Zipper” library system. When applied to screen against the HCV G4 RNA, they successfully obtained active compounds with a binding affinity of 10.9 μM [130], demonstrating the potential of DEL technology for targeting structured RNA.

**Fig. 8:**
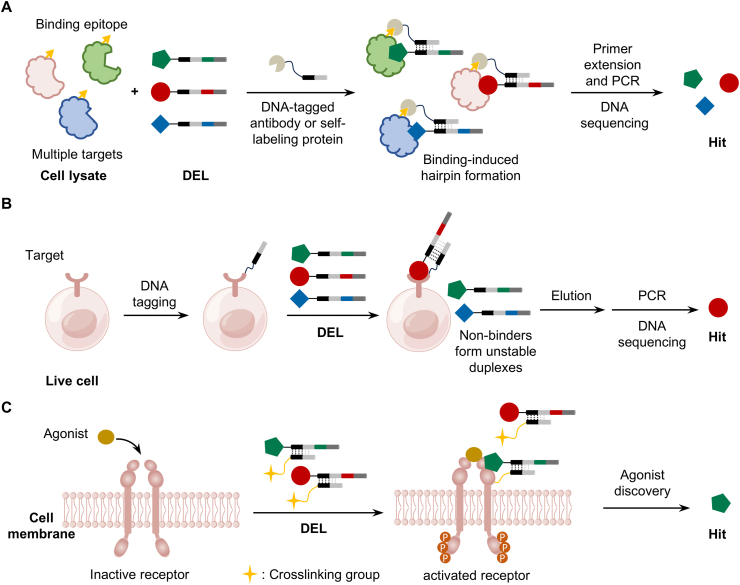
The crucial selection strategies (A) Interaction determination using unpurified proteins (IDUP) selection method in cell lysates; (B) DNA-programmed affinity labeling (DPAL)-driven DEL selection against membrane protein targets on live cell surfaces; (C) Identifying insulin receptor (INSR)-specific agonists using natural protein agonist-guided DEL selection.

Regarding selection environments, DEL technology has achieved a transition from purified systems to complex biological systems like cell lysates [168] and live cells [[99], [100], [101]] (Fig. 8). Liu et al. performed DEL selection against unpurified proteomes in cell lysates and successfully discovered a series of covalent inhibitors for kinase targets, validating the feasibility of direct screening in near-physiological environments [169]. What is particularly ingenious is that Li and colleagues established a live cell-based selection platform. This method enables target-specific DEL selections against endogenous membrane proteins on live cells by specifically labeling these proteins with a DNA tag, eliminating the need for overexpression or any genetic manipulation. The feasibility and utility of this strategy were successfully validated using targets such as the FR, CA12 and EGFR [99]. Building on this, the Li team further developed a live-cell screening platform. Through directed screening against the membrane protein insulin receptor (INSR) on live cells, they identified novel agonists with sub-nanomolar affinity and low micromolar cellular activity, marking the entry of DEL screening into a new stage of functional selection [101]. Looking ahead, this evolution from solution to cellular contexts is expected to branch into more refined strategies. The logical progression would be toward achieving subcellular precision, such as organelle-specific (e.g., the mitochondria, or endoplasmic reticulum) or site-specific (e.g., the allosteric site) selection.

The synergistic development of these selection strategies has collectively driven the strategic transformation of DEL technology from traditional affinity-based screening to function-oriented selection. By establishing more physiologically relevant screening environments and expanding the range of targets, DEL technology provides a powerful platform for ligand discovery against challenging drug targets, significantly accelerating the process of innovative drug discovery.

### 3.4 Integration with AI

The deep integration of DEL technology with AI is reshaping the paradigm of drug discovery. Leveraging its core strength in rapid construction and screening of large-scale molecular libraries, DEL achieves breakthroughs in traditional limitations through AI synergy: On one hand, AI algorithms effectively analyze massive datasets generated by DEL screening, utilizing machine learning models to reduce background noise interference and significantly enhance hit rates of active compounds. On the other hand, the real-world screening data provided by DEL serves as a high-quality dataset for AI training, creating a mutually reinforcing ecosystem. For instance, Riley’s team trained machine learning models using DEL data, successfully identifying novel sEH/ERα/c-KIT inhibitors from commercial libraries that were not derived from DEL [170]. Similarly, Feng et al. discovered compounds with structurally distinct profiles from DEL library members based on CA IX target DEL data [171]. The Lu group further demonstrated precise prediction of BRD4/p300 active molecules by integrating affinity/photoaffinity selection data [172]. Beyond data analysis, AI also revolutionizes DEL construction, as exemplified by Hou and Xu et al., who employed machine learning to predict high-yielding, selective SeNEx reactions. This approach boosts on-DNA chemistry success, expands accessible chemical space, and ultimately enhances library quality and screening efficiency [173]. These practices substantiate that AI-DEL integration strategies not only accelerate the discovery of lead compounds but also expand structural diversity through data-driven approaches, continuously propelling drug research toward greater efficiency and intelligence.

## 4 Conclusion and perspective

DEL technology has revolutionized early-stage drug discovery by enabling rapid and cost-effective selection of ultra-large chemical spaces. This review systematically summarizes the remarkable progress of DELs in addressing a wide range of challenging targets, including GTPases, epigenetic targets, phosphatases, protein-protein interactions, membrane proteins, and RNA. Through innovative library design, advanced selection strategies, and integration with AI, DEL has demonstrated its unique capability to identify high-affinity ligands for targets previously considered “challenging”. To overcome the next generation of drug discovery challenges and fully realize the potential of these novel ligands, the advancement of DEL technology will be propelled by three key, interconnected frontiers.

The first is the foundational expansion of the chemical and methodological capabilities of DEL. This encompasses the ongoing development of DNA-compatible chemistry, especially for introducing chirality, sp^3^-hybridized carbons, and complex privileged scaffolds, which is essential for enhancing structural three-dimensionality and drug-likeness. Concurrently, establishing rigorous standards to evaluate true DEL compatibility, beyond mere DNA compatibility, is critical. This requires developing new experimental paradigms to ensure chemical reactions are robust in combinatorial matrices and efficient on fully assembled DNA barcodes for reliable library production. The second frontier focuses on the strategic design and quality control of libraries to improve screening outcomes. This includes constructing functionally specialized libraries tailored to specific target classes or mechanisms, thereby focusing resources on the most relevant chemical space. Moreover, as libraries grow in scale and complexity, ensuring their integrity presents a major challenge. Future efforts must advance beyond analyzing model compounds to create robust analytical or predictive frameworks that guarantee the fidelity of mega-libraries throughout synthesis, storage, and screening. The third frontier involves paradigm-shifting integrations with functional biology and data science. The convergence of DEL with functional selection methods, such as cellular phenotyping and targeted protein degradation assays, will move discovery from affinity-based screening to direct function-driven interrogation. Simultaneously, the deep integration of AI will transform data analysis, enabling intelligent mining of vast selection datasets to decipher complex SAR and accelerate the progression from initial binders to novel lead candidates.

In summary, this review not only provides a comprehensive and up-to-date survey of DEL applications across established challenging target classes (e.g., GTPases, PPIs, phosphatases) but also makes a distinct contribution by systematically consolidating the rapidly advancing field of DEL-based RNA-targeted hit identification, a key frontier in modern drug discovery. We also illustrate how the convergence of novel library architectures, including covalent, macrocyclic, and RNA-targeted designs, with innovative live-cell selection strategies and artificial intelligence is transforming DEL from a conventional screening method into a multifaceted discovery engine. By highlighting these strategic advances and their successful translation into chemical probes and leads against historically intractable targets, we hope this work can offer a forward-looking perspective on DEL’s expanding role in drugging the undruggable and shaping the future of therapeutic discovery.

## CRediT authorship contribution statement

**Nana Du:** Writing – review & editing, Writing – original draft, Visualization. **Yijun Li:** Visualization. **Qing Lou:** Visualization. **Gong Zhang:** Writing – review & editing, Supervision, Funding acquisition. **Yangfeng Li:** Writing – review & editing, Supervision, Funding acquisition. **Yizhou Li:** Writing – review & editing, Project administration, Funding acquisition, Conceptualization.

## Ethics approval

Not applicable.

## Declaration of generative AI in scientific writing

No generative AI tools have been used throughout the entire writing process of this manuscript.

## Funding

This work was supported by grants from the 10.13039/501100001809National Natural Science Foundation of China (nos. 22361162605, 22477011, 22107017, and 22222702) the Scientific Research Innovation Capability Support Project for Young Faculty (ZYGXQNJSKYCXNLZCXM-H24), and the 10.13039/501100005230Natural Science Foundation of Chongqing (nos. CSTB2025NSCQ-LZX0009 and CSTB2024NSCQ-MSX0675).

## Declaration of competing interest

The authors declare the following financial interests/personal relationships which may be considered as potential competing interests: Yizhou Li reports financial support was provided by National Natural Science Foundation of China. Yangfeng Li reports financial support was provided by National Natural Science Foundation of China. Gong Zhang reports financial support was provided by National Natural Science Foundation of China. Yizhou Li reports financial support was provided by Scientific Research Innovation Capability Support Project for Young Faculty. Yizhou Li reports financial support was provided by Natural Science Foundation of Chongqing. Yangfeng Li reports financial support was provided by Natural Science Foundation of Chongqing. If there are other authors, they declare that they have no known competing financial interests or personal relationships that could have appeared to influence the work reported in this paper.

## Data Availability

Not applicable.
